# Development of the “Highly Sensitive Dog” questionnaire to evaluate the personality dimension “Sensory Processing Sensitivity” in dogs

**DOI:** 10.1371/journal.pone.0177616

**Published:** 2017-05-16

**Authors:** Maya Braem, Lucy Asher, Sibylle Furrer, Isabel Lechner, Hanno Würbel, Luca Melotti

**Affiliations:** 1 Division of Animal Welfare, Veterinary Public Health Institute, Vetsuisse Faculty, University of Berne, Berne, Switzerland; 2 Centre for Behaviour and Evolution, Institute of Neuroscience, Newcastle University, Newcastle, United Kingdom; 3 Veterinary Public Health Institute, Vetsuisse Faculty, University of Berne, Berne, Switzerland; University of Pisa, ITALY

## Abstract

In humans, the personality dimension ‘sensory processing sensitivity (SPS)’, also referred to as “high sensitivity”, involves deeper processing of sensory information, which can be associated with physiological and behavioral overarousal. However, it has not been studied up to now whether this dimension also exists in other species. SPS can influence how people perceive the environment and how this affects them, thus a similar dimension in animals would be highly relevant with respect to animal welfare. We therefore explored whether SPS translates to dogs, one of the primary model species in personality research. A 32-item questionnaire to assess the “highly sensitive dog score” (HSD-s) was developed based on the “highly sensitive person” (HSP) questionnaire. A large-scale, international online survey was conducted, including the HSD questionnaire, as well as questions on fearfulness, neuroticism, “demographic” (e.g. dog sex, age, weight; age at adoption, etc.) and “human” factors (e.g. owner age, sex, profession, communication style, etc.), and the HSP questionnaire. Data were analyzed using linear mixed effect models with forward stepwise selection to test prediction of HSD-s by the above-mentioned factors, with country of residence and dog breed treated as random effects. A total of 3647 questionnaires were fully completed. HSD-, fearfulness, neuroticism and HSP-scores showed good internal consistencies, and HSD-s only moderately correlated with fearfulness and neuroticism scores, paralleling previous findings in humans. Intra- (N = 447) and inter-rater (N = 120) reliabilities were good. Demographic and human factors, including HSP score, explained only a small amount of the variance of HSD-s. A PCA analysis identified three subtraits of SPS, comparable to human findings. Overall, the measured personality dimension in dogs showed good internal consistency, partial independence from fearfulness and neuroticism, and good intra- and inter-rater reliability, indicating good construct validity of the HSD questionnaire. Human and demographic factors only marginally affected the HSD-s suggesting that, as hypothesized for human SPS, a genetic basis may underlie this dimension within the dog species.

## Introduction

Research on individual differences and, hence, differing “personalities” in animals has increased considerably in recent years [1–6], although in animal research, the terms “personality”, “temperament”, and “behavior” are often used interchangeably. There is no unanimous definition of the term “personality” in humans or animals, nevertheless, it can be broadly described as a set of behavioral and physiological characteristics that are consistent across time and different contexts [2,5,7–12]. It has been proposed that personality may contribute to the maintenance of behavioral variation and thus allow for adaptive responses to changes in the environment [13–15]. The most studied personality dimensions in animals include exploration [16], boldness-shyness [9,17–24], responsiveness to variation in the environment [25–27], fearfulness [16,28], and aggression [16].

Some animal personality research has investigated the extent to which animals share personality dimensions analogous to those found in humans [10,29,30]. Human models of personality have been quite successfully translated to animals. Gosling and John [30], for example, reviewed 19 studies investigating personality in 12 nonhuman species. Based on one of the most popular models of human personality, the Five-Factor-Model [31,32], they found that certain dimensions—namely extraversion, neuroticism, agreeableness, and, to a lesser degree, openness—were present in all animal species investigated, while others (e.g. conscientiousness and “level of activity”) were only found in some species. Thus, whether human personality dimensions translate to animals depends both on the dimensions and the species studied.

In companion animals, the assessment and understanding of personality has important implications for the welfare of both pets and owners, and may facilitate prevention and treatment of behavioral problems by animal behavior specialists. Research in this field has mainly focused on the selection of appropriate individuals for specific types of work, such as dogs for the blind or partially sighted, search dogs or police dogs [33–35], on the measurement of specific personality dimensions by means of tests [9,36] and questionnaires [37,38], and on similarities found in human and animal personality [39–41]. Less attention has been paid to whether individuals with different personalities may respond differently to varying training or communication types, or whether they may be more prone to develop certain behavior problems. There is increasing evidence in the human literature of the interplay between personality and environmental influences in affecting susceptibility to stress [42–48], the development of mental and physical problems [42,43,49–55] and the responsiveness to treatment approaches [56].

A relatively new personality dimension in humans referred to as “sensory processing sensitivity” (SPS), or “high sensitivity”, was first described by Aron and Aron in 1997 [57]. These authors define SPS as “a genetically determined dimension involving a deeper […] cognitive processing of stimuli that is driven by higher emotional reactivity” [58]. SPS is not to be confused with “sensory processing sensitivity disorder”, which is a pathological syndrome [59]. SPS is hypothesized to have a genetic component, the phenotypic expression of which is influenced by the interaction with (pre-, peri-, and postnatal) environmental conditions [60]. It involves a deeper processing of thought and emotions, greater likelihood of being overaroused, higher emotional intensity (both positive and negative) and higher sensitivity to subtle stimuli perceived by all modalities, i.e. visual, acoustic, tactile, olfactory, gustatory, proprioceptive [58,60–62]. These characteristics of SPS have been suggested to be a consequence of deeper and more complex cognitive processing, and not to be linked to an actual higher acuity of the sense organs [60]. Different levels of SPS have been shown to be linked to differences in the dopaminergic neurotransmitter systems [63] and in the serotonergic system, with parallels suggested between high SPS and the s-allele of the serotonin transporter linked polymorphic region (5-HTTLPR) [62]. Several fMRI studies have also shown differences in brain functioning between individuals scoring higher and lower on SPS. Individuals scoring high on SPS showed less cultural differences in the judgment of visual stimuli [60], higher activation of brain regions linked to awareness, empathy, integrating and distinguishing own emotions from other’s, and of the mirror neuron system in the face of social affective stimuli, i.e. in response to photos of their partner’s or a stranger’s happy, sad and neutral faces [61,64]). Jagiellowicz and colleagues found higher activation of brain areas involved in high-order visual processing and of the right cerebellum when confronted with images with subtle changes [65]. These findings suggest the existence of a physiological basis for the differences in perceiving, processing and responding to information shown by highly sensitive individuals.

Therefore, SPS can influence the manner in which people process information in their environment and, hence, the interaction between the environment and SPS can affect their psychological wellbeing [66–68]. With respect to animal welfare, it is thus important to know whether this personality dimension translates to animals. If it does exist in other species, it might influence how these animals perceive and process information. Depending on the environment in which these animals live, this in turn would affect their wellbeing at the individual level.

Whilst much research has focused on finding analogues of human personality in animals, the SPS personality dimension has not been studied in animals to date. There are, however, reasons to believe that such a dimension could exist in animals as well. Dichotomous ways of reacting in the face of novel or stressful stimuli have been described in several animal species [69]. These include, for example, proactive and reactive coping styles in rodents [11,70] and pigs [71,72], fearful / uptight and not fearful / not uptight rhesus macaques [73], and slow versus fast exploring, or shy versus bold, great tits [22,74–76]. Additionally, already existing questionnaires looking at dog personality contain elements that might overlap with SPS, e.g. the excitability or touch sensitivity sub-scores found in the C-BARQ questionnaire [77]. It is possible that these response types may be associated with the SPS dimension; however, to what extent they overlap with, differ from, or coincide with a response strategy of highly sensitive individuals in similar situations remains to be determined.

In the attempt to identify and explore SPS in companion animals, pet dogs represent perhaps the ideal subjects for investigation. In recent years several studies have investigated dog personality, providing a series of tools (e.g. questionnaires) for its assessment [9,37,41,78–81] and also critical views on the types of assessment available [82]. Moreover, dogs have already been used as models to study the regulating mechanisms of human behaviour [83,84]. Hence, this species lends itself as a starting point to investigate this personality dimension in animals. In dogs and companion animals in general, personality is frequently assessed using questionnaires completed by a person who knows the animal well [85]. However, before a questionnaire can be used reliably, its internal and external validity need to be assessed, e.g. to prevent issues related to subjectivity and anthropomorphism [86].

To this end, we developed a “highly sensitive dog” (HSD) questionnaire to identify and evaluate a possible “canine sensory processing sensitivity” personality dimension (cSPS). Questions were initially selected in a pilot study, and the HSD questionnaire was then validated by means of an international, large-scale online survey. We hypothesize that a) a SPS personality dimension (cSPS) similar to the one described in humans can be measured in dogs using the HSD questionnaire; b) cSPS will be partly independent from the personality dimensions fearfulness and neuroticism as previously found in humans, whereby the construct of neuroticism, defined in humans as the propensity to experience intense negative emotions [87], has not been studied in detail in animals [61,88]; and c) the variability of the HSD score will be explained to a small degree only by demographic and human factors, such as breed, sex, previous experience, human interaction, and environmental factors known to affect behavior, suggesting that a genetic basis within *canis familiaris* may underlie the cSPS personality dimension.

## Exploratory pilot study—Development of the HSD questionnaire

### Materials and methods

#### Questionnaire development

The development of the Highly Sensitive Dog (HSD) questionnaire presented two main challenges. First, the SPS dimension had not been previously described in these terms in animals, hence there was no previous research on SPS in dogs to refer to. Second, as several questions from the validated Highly Sensitive Person (HSP) questionnaire were not applicable to animals (e.g., “I am deeply moved by the arts and music”), it was not possible to extrapolate the complete validated HSP questionnaire for humans [57] directly to dogs. Therefore, a qualitative interview approach was adopted based on the methodology used to develop the HSP questionnaire for humans by Aron and Aron [57]. This entailed an initial qualitative approach (pilot study) which provided the basis for a subsequent quantitative large-scale survey aimed to confirm and support the preliminary findings of the pilot study.

In their first work on SPS, Aron and Aron [57] published an advertisement asking people who considered themselves to be highly sensitive to contact them for open interviews. Similarly, in the present study, owners of dogs considered to be highly sensitive were selected and interviewed. The HSD questionnaire was then developed based on the information collected from these interviews, which was combined with relevant parts of the already existing Highly Sensitive Person (HSP) and Highly Sensitive Child (HSC) questionnaires [57,89], and of other dog personality questionnaires detailed below [79–81].

#### Interviews to identify descriptors potentially related to cSPS

Fifteen dog owners from the region of Basel, Switzerland, were contacted, whose dogs were considered to be highly sensitive based on the SPS definition in the human literature [57]. Dog owners were selected by the first author and a dog trainer based on direct observation of the dog (N = 14), with dogs showing behaviors such as attention to detail, picking up on owner’s emotional states, being very attentive, showing “stop and watch” behaviors when faced with new situations / objects, and by posting an advert on the University of Basel website to recruit any dog owners who thought their dogs might be highly sensitive (N = 1). All participating dog owners gave their informed consent of participation.

The owners underwent one open interview of approximately 2 hours’ duration with the first author. Interviews were carried out over a two-month period and consisted of questions regarding the dog’s history, first and current living conditions, health, and behavior (e.g. towards people and other dogs, in crowded places, when stressed, etc.). Dog owners were also asked to describe their dog’s personality, whether they thought their dog was highly sensitive, and, if so, what made them believe this was the case.

The interviews led to the development of a list of “descriptors”, i.e. brief sentences describing the dog’s personality, such as “my dog observes a lot” or “my dog reacts strongly to smells” or “my dog is very cuddly”. These descriptors were combined with those questions of the HSP questionnaire and those of a not yet validated version of this questionnaire for children (the Highly Sensitive Child (HSC) questionnaire [89], which could be extrapolated to dogs (please see S1 Appendix for origin of the questions). The HSC questionnaire was used, as for both young children and dogs, questions can only be answered by proxy (i.e. by the parent and the owner, respectively), and because the questions were already formulated in a way that allowed easier extrapolation to dogs.

Furthermore, elements of four questionnaires already in use to evaluate dog personality were included as well, namely the Monash Personality Questionnaire [80], the Canine Big Five Inventory (C-BFI) [38,90], the positive and negative activation score (PANAS) [81], and an impulsivity questionnaire developed by Wright and colleagues [79]. These questionnaires were chosen because they included personality aspects which were expected to at least partly overlap with cSPS (e.g., reaction to stressful situations, perception of subtleties in the environment, sensitivity to sensory information, impulsivity, etc.) and could also serve to distinguish cSPS from other personality dimensions such as neuroticism (C-BFI, Monash) and fearfulness (PANAS). Questions that were formulated similarly in two or more questionnaires were combined and only included once in order to shorten the questionnaire and to avoid repetition. This led to the wording of combined questions to not always be literally adopted from the other questionnaires. For instance, Question 15 of the HSC-Q, Questions 18 and 25 from the HSP-Q, and Question 34 from the C-BFI are all represented in the question “my dog reacts when we argue at home”. The described procedure led to 112 questions in total (see S1 Appendix).

### Choice of questions for the HSD questionnaire

The 112 questions referring to the dog’s personality, together with the validated 27-item HSP questionnaire to assess SPS of the owners [57], were combined in an online questionnaire which was sent to two groups of dog owners: a presumed highly sensitive dog group (HSD-Group) and a presumed non-highly sensitive dog group (nHSD-Group). The HSD-Group consisted of 16 owners of dogs which were considered to be highly sensitive by the first author based on the definition of SPS in humans. Thirteen of these dog owners had participated in the previous open interviews, while the remaining three were recruited by the first author and the collaborating dog trainer. The nHSD-Group consisted of 10 owners of dogs which were considered not to be highly sensitive, as they did not fulfil the criteria for high sensitivity described for humans. The questions were presented in German and formulated as statements (S1 Appendix). With the first author (MB) and two other co-authors (SL and SF) being bilingual in German and English, the translation of the questionnaire from the original German version into the English version was done by the first author, while the two other co-authors checked the accuracy of the back-translation into German. The owners were asked to reply on a 7-point likert scale (1 = does not apply at all, 7 = applies completely). The HSP questionnaire was completed by 14 owners from the HSD-Group, and by 9 owners from the nHSD-Group.

### Data analysis

Statistical analysis was performed using SPSS Version 21. Where data did not meet parametric assumptions and transformations were not effective, non-parametric tests were used. Scores obtained from negatively worded questions (N = 15) were inverted so that a high score always indicated supposed high sensitivity for all questions. One mean score per dog was calculated by averaging the scores of all 112 questions. The difference between the mean score of the HSD- and nHSD-Groups was assessed using an independent samples t-test.

Also, absolute means were calculated for every question. For every question, the difference between the means of both groups (i.e., HSD–nHSD) was calculated and ranked from largest to smallest. To determine which questions differentiated between the HSD and nHSD groups, Mann Whitney U tests were performed for each question in sequence of the questions ranked according to the difference of means between the two groups. Questions where this difference was significant to p < 0.05 were selected for further consideration in the final HSD questionnaire. Furthermore, when these differences approached significance (p < 0.07), only the questions that directly originated from one of the three major subcategories identified in human SPS (i.e., “ease of excitation”, “aesthetic sensitivity” or “low sensory threshold” [88]) were selected for the final HSD questionnaire.

Cronbach’s alpha was calculated for the selected questions to assess their internal consistency.

Relationships between the mean HSP score (HSP-s) of the owners and both the mean HSD-score and the mean nHSD-score of the dogs were assessed using Spearman’s rank correlations. No corrections for multiple testing were applied in this exploratory pilot study.

### Results

Information on breed, age and sex of the dogs participating in the pilot study is shown in Table 1.

**Table 1:** Demographics of the dogs participating in the pilot study.

	Pilot Study Interviews (N = 15)	Pilot Study HSD* Group (N = 16)	Pilot Study nHSD** Group (N = 10)
**Breeds**	4 Australian Shepherds	4 Australian Shepherds	1 Border Collie
	2 Duck Tolling Retrievers	2 Duck Tolling Retrievers	1 Eurasian
	2 Mixed breeds	5 Mixed breeds	4 Mixed breeds
	1 Flatcoated Retriever	1 Flatcoated Retriever	1 Flatcoated Retriever
	1 Labrador Retriever	1 Labrador Retriever	1 Irish Terrier
	1 Lagotto Romagnolo	1 Lagotto Romagnolo	1 Sheltie
	1 Scottish Terrier	1 Scottish Terrier	1 Scottish Terrier
	1 Miniature Poodle	1 German Shepherd	
	1 Tibet Terrier		
	1 Vizla		
**Age** in years (Median & Range)	4.8 (0.5–10)	5.3 (0.5–10.3)	5.9 (1.4–12.8)
**Sex**	5 male intact	8 male intact	2 male intact
	8 male neutered	4 male neutered	5 male neutered
	1 female intact	3 female intact	3 female neutered
	1 female neutered	1 female neutered	

* HSD = highly sensitive dog;

** nHSD = not highly sensitive dog.

Mean scores (based on all 112 questions) of the HSD Group (M = 4.64, SD = 0.46) were higher than mean scores of the nHSD Group (M = 3.94, SD = 0.29; t(24) = 4.35, p < 0.001).

Mann Whitney U tests comparing mean scores of each question between the two groups showed that 32 of the 112 questions met the selection criterion for inclusion in the final HSD questionnaire.

Cronbach’s alpha of the 32 selected questions was 0.8 for the HSD group and 0.7 for the nHSD group. No significant correlations were found between the mean scores (based on the 32 selected questions) of either the HSD or nHSD group and the HSP-s of the owners (r_s_ = .45, p = 0.11; r_s_ = .39, p = 0.31; respectively).

A list of the 32 selected questions forming the HSD questionnaire, including the original sources of each question, can be found in S2 Appendix.

## Main study: International online survey

### Material and methods

In order to further develop and validate the HSD questionnaire of the pilot study, a larger international online study was performed. This online survey included the developed HSD questionnaire (32 questions) and eight additional questions. These additional questions were added to assess if and to what extent the final HSD-s overlapped with the personality dimensions of fearfulness (N = 7; Questions 4, 5, 13, 20, 37, 41, 42; [34,81]) and neuroticism (N = 2; Questions 10, 41; [80]; please note that Question 41 applied for both neuroticism and fearfulness dimensions). The additional questions regarding fearfulness were added based on questions in the PANAS questionnaire [79] and on four questions identified by Harvey and colleagues (Questions 5, 13, 20, 37) [34], who labeled them as “general anxiety” and found them to correspond well with the personality dimension “fearfulness” described by Jones et al. [82]. Hence the fearfulness score was made up of the mean of the seven questions that were additionally added. The questions relating to neuroticism were based on the adjectives that make up the neuroticism dimension in the Monash questionnaire [78]. Four of the 32 questions of the HSD questionnaire already overlapped with these adjectives (Questions 3, 6, 9, 32). Two questions were added to complete the neuroticism dimension according to the Monash questionnaire (Questions 10 and 41). Hence, the final neuroticism score consisted of the mean of six questions, two of which were not part of the 32-item HSD questionnaire. All the questions were formulated as statements and replied to on a 7-point likert scale (1 = does not apply at all, 7 = applies completely). The questionnaire was filled in by the owner who was defined as “the person who has the closest relationship with the dog”. In order to investigate the dependency of the HSD-s on external factors, general questions that have been discussed in the literature to influence behavior [12,91] were included, such as owner sex, age, and profession, dog breed, age, and sex, questions regarding the dog’s history and current living situation, the dog’s health, the presence of behavior problems, types of training methods (positive and negative punishment and positive reinforcement) used by the owner, time spent with the dog, as well as the (subjective) stimulation levels of the first and current living surroundings (see Table 2 for the complete list). Based on the assumption that certain types of “milder” positive punishment are more likely to be used than stronger ones, positive punishment was separated into mild (e.g. using the voice or slight touch) and strong (i.e. using harsh physical methods, such as hitting or shock collar). Negative reinforcement was not included, as it was not possible to formulate questions appropriately in the questionnaire. The human HSP questionnaire was also included to investigate whether the owner personality related to the personality assessment of the dog. In order to assess whether the degree of knowledge on dog behavior had any effect on the HSD-s, the participants were asked to indicate whether they belonged to one or more professional categories (see Table 2). The questionnaire was made available in English and German (see S3 Appendix for the general questions of the online survey in English).

**Table 2:** Factors included in the linear mixed models with the outcome variable being the highly sensitive dog score (HSD-s).

	Factor	Unit / Options
**Demographic factors**	Dog sex	male intact, male neutered, female intact, female neutered
	Dog weight	kg
	Dog age	years
	Dog age at adoption	months
	Country of origin	same from now, different from now
	Previous owner	Yes, no, I don’t know
	Number of people in household	1, 2, >2
**Human factors**	Owner age	< 18 years; 19–30 years; 31–65 years; > 65 years
	Profession of owner	dog trainer; veterinarian; behavior specialist; university student; university employee; none of the above
	Type of Communication	R+: e.g. food treats, cuddle; clicker/marker word, praise with voice, play, etc.
		P+ strong: e.g. turn on back, press dog to ground, tug on lead, choke collar, kick, etc.
		P+ mild: voice (shout, sharp), obedience work, spray collar, hand over muzzle, noise interrupter (discs), spray with water, tap on nose
		P-: withhold reward, time-out, ignore
	Current degree of stimulation in surroundings	Likert scale 1–5 (5 being the highest degree of stimulation)
	Active time per day	< 1 hour; 1–3 hours; > 3 hours; I don’t know
**Random effects**	Country in which the dog currently lives	Switzerland, Germany, Austria, UK, USA, Canada, other
	Breed	Open text; post hoc categorization into Fédération Cynologique Internationale (FCI) groups

### Participants

Participants were recruited by sending out emails to universities, organizations, and professional contacts, by distributing information on facebook and printed flyers in veterinary offices, at veterinary hospitals, conferences, pet shops, and professional contacts in the German-speaking countries Switzerland, Germany, and Austria and the English-speaking countries the United Kingdom, the United States of America, and Canada. The survey was created with the software Limesurvey v2.05 and remained online for 2.5 months from mid-October to the end of December 2014.

Intra- and inter-rater reliability were assessed by sending an email to all participants who had provided their email addresses (N = 2804) six months after the first questionnaire had been completed. This email included the request for the same person (i.e. the person referred to as “the owner” in this manuscript) to fill in the same questionnaire for the same dog again (to assess intra-rater reliability). The owner was additionally invited to ask another person who knew the dog well to fill in the questionnaire for the same dog (to assess inter-rater reliability). Due to the large number of participants, further inclusion or exclusion criteria with regard to the choice of the second person filling in the questionnaire were not possible. At the point in time of the second evaluation however, all owners had had their dogs for at least 6 months, due to the time period between filling in the first and second questionnaire.

### Data analysis

Statistical analysis was performed using R software version 3.02 (R Core Team, 2013). Due to the large sample size and potential for over-power, the effect sizes in this study were considered to be more informative than p-values.

For each dog, a mean HSD-s, fearfulness score (based on seven questions), neuroticism score (based on six questions) and for each owner a mean HSP-s were calculated. Internal reliabilities of the questions referring to cSPS, neuroticism, fearfulness, and SPS were measured using the Cronbach’s α test, where values for alpha greater than 0.7 were considered acceptable. Since the questionnaire was available in two languages, Cronbach’s alphas were calculated for each language and for both languages combined. A linear model (command lm) was used to explore language differences (as fixed effect) in HSD-s (outcome variable). A two-sample permutation t-test (implemented in R package Deducer using the perm.t.test command) was run for HSD-s and HSP-s to compare the responses of male and female owners, whilst controlling for the bias toward female respondents. Fearfulness, Neuroticism, HSD-s, and HSP-s were tested to see if they approximated a normal distribution using the Kolmogorov–Smirnov test and consideration of skewness and kurtosis values. In order to test whether cSPS was related to neuroticism and/or fearfulness, correlations amongst these dimensions were tested using Spearman’s rank correlation. This analysis was necessary to test construct validity of the new cSPS dimension, where a positive yet moderate correlation between the dimensions was expected. Intra-rater and inter-rater reliability were tested using correlations and Bland Altman plots and statistics. The *a priori* criteria set for accepting reliability were a correlation of 0.6 or above and less than 5% of participants outside the critical limits in the Bland Altman plots. Due to the timing between the first and second completion of the questionnaire, intra-rater reliability also provided information on consistency of behavior over time.

In order to test whether the cSPS dimension was independent of other factors (which were labeled “demographic factors” and “human factors”; see Table 2 for details) as hypothesized, the influence of these factors on the variance of HSD-s was tested using linear mixed effect models. Forward stepwise selection (implemented using the R package lme 4.0 and the code lmer) was used, with demographic and human factors tested as fixed effects (listed in Table 2). In order to understand the different impacts of demographic and human factors and because of correlations between these groups of effects, two separate models were built, one for demographic factors and one for human factors. In both models, dog breed and country were included as separate random effects (there was no interaction between country and breed on HSD-s), and HSD-s was the outcome variable. Once each model had been built, a pseudo R-squared value was calculated to estimate the amount of variance explained by the explanatory variables in the model. The R package MuMIn and the command r.squaredGLMM were used to produce R squared values for the fixed effects alone and the fixed and random effects together. Finally, to understand the effects of human sensitivity on ratings of dog sensitivity, a liner model (command lm) was used to analyze the relationship between HSD-s and HSP-s. R- squared values are reported.

A principal components analysis on the c-SPS questions was performed to explore the possibility of the existence of subtraits within the trait c-SPS. The command principal was used from the psych package, and scree plots and eigenvalues were used to select the number of components to report. Bartlett's sphericity test and the KMO index were checked on the final PCA which used a covariance matrix structure and applied varimax rotation to the loadings. The loadings are presented with the highest modal loading value used to decide which component each question was placed within.

### Results

#### Descriptive statistics

A total of 3647 fully completed questionnaires were returned (56% of the total replies) and analyzed. Thirty-four percent of the replies came from German-speaking countries (Switzerland: 21%, Germany: 12%, Austria: 1%), 47% from English-speaking countries (USA: 23%; UK: 16%; Canada: 8%), and the remaining from other countries not further specified. As responses from Austria only made up 1% of the total, these were grouped in the “not further specified” countries.

**Fig 1:**
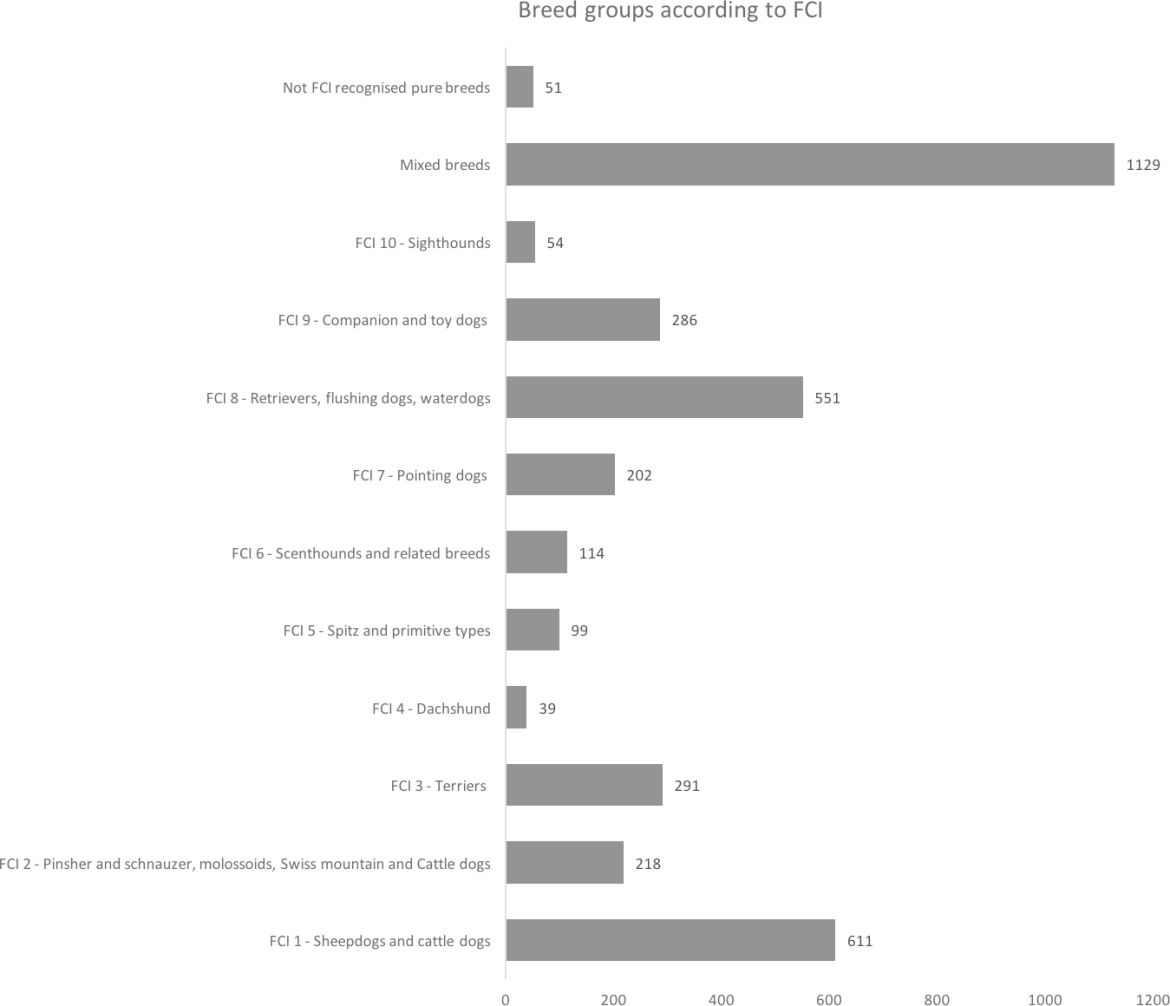
Distribution of participating dog breeds according to the Fédération Cynologique International (FCI). Numbers behind bars represent frequencies.

The dog breeds participating in the study were grouped according to the Fédération Cynologique International (FCI) breed groups, with addition of the groups “breeds not recognized by the FCI” and “mixed breeds” (Fig 1)

Dog sex was evenly distributed with 50% males (of which 68% were neutered) and 50% females (of which 76% were neutered). The mean age of dogs was 5.8 years (SD = 3.6) and the mean weight 21.3 kg (SD = 11.7).

The majority of the dogs had been adopted within the same country in which they currently lived (85%). For 65% of the dogs, the current owner was the first owner to adopt them from the birth place, 26% had had at least one previous owner, 9% of the owners indicated “I don’t know”. In 49% of the households there were two people, in 33% there were more than two people, and in 18% there was only one person.

Ninety-one percent of the owners were women and 9% men. The majority of the owners were aged between 31–65 years (69%), 26% were between 18–30 years of age, the remaining 5% were either younger or older.

As the professional group of behavior veterinarians was very small (1%), it was combined with the group “dog trainer specialized in behavior” for further analysis based on the assumption that these two groups had the best knowledge of dog behavior. The group “university employee” was quite small as well (6%), hence it was combined with the group “university student”. If a person belonged to two categories, the one of higher supposed significance for understanding canine behavior was chosen, i.e. behavior trainer and/or behavior veterinarian (7%) > dog trainer (10.6%) > veterinarian (4.8%) > university employee / student (16.9%) > none of the mentioned categories (60.8%). Behavior veterinarians and behavior trainers both have a specialized further education in canine behavior. As the degree of practical knowledge regarding behavior tends to be low in veterinarians [92,93] and dog trainers work with dog behavior on a daily basis, it was decided to rank dog trainers as having more knowledge regarding canine behavior than veterinarians.

As all owners but three used positive reinforcement, this category was not considered in further analysis. Three percent of owners only used strong positive punishment, 6% used a combination of strong and mild positive punishment, 19% only used mild positive punishment, 19% only negative punishment, and 13% used all types of punishment. A large proportion of owners (33%) used a combination of positive reinforcement, mild positive punishment and negative punishment techniques.

Most of the dogs (39%) lived in surroundings with a medium degree of stimulation as estimated by their owners on a 5-point likert scale (score of 3), 12% with very little (score of 1) and 12% with a lot of stimulation (score of 5). Slightly more (21%) lived in surroundings with low to medium stimulation (score of 2) than did in surroundings with medium to high stimulation (score of 4; 16%).

Most of the owners (70%) spent between 1–3 hours a day of active time with their dogs, 14% less than an hour, and 16% more than 3 hours.

#### HSD, fearfulness, neuroticism, and HSP scores

For the dogs, the HSD-s ranged from 1.41 to 6.74 with a mean of 4.03 (SD = 0.9), the fearfulness score ranged from 1.15 to 6.88, with a mean of 4.07 (SD = 1.26), and the neuroticism score ranged from 1.21 to 6.89 with a mean of 4.11 (SD = 1.27). The HSP-s for the owners ranged from 1.41 to 7 with a mean of 4.20 (SD = 1.09). The distributions of the HSD-s and neuroticism score were close to normal. The HSP-s and fearfulness score were not good approximations to the normal distribution. HSP-s was left-skewed (skewness = 0.44), which suggests the presence of a longer tail/spike on the right side where scores are high.

#### Intra- and inter-rater reliability

**Fig 2:**
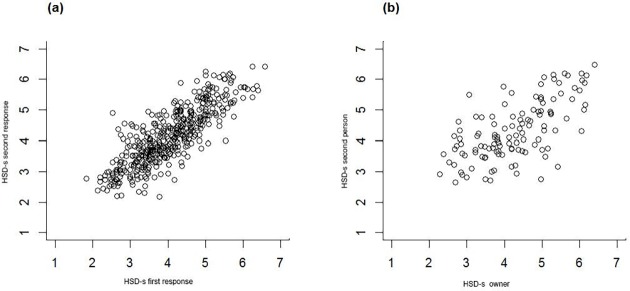
**Association between HSD-s for the same dog recorded (a) by the same person 6 months apart (intra-rater), and (b) by two different persons knowing the same dog well (inter-rater)**.

Six months after completion of the first questionnaire, a total of 447 owners (intra-rater reliability) and a total of 120 other people who knew the same dog well (inter-rater reliability) filled in the questionnaire for the same dog. cSPS met the criteria set for both intra- and inter-rater reliabilities. The correlation between HSD-s on the first occasion and after 6 months was very high (r = .83, p < 0.001; Fig 2A). The Bland Altman plots and statistics indicated that people rarely rated dogs more than one point different on the scale despite completing assessments six months apart (3%, i.e. 15 out of 447 people rated outside the critical difference of 1.05). The mean difference between first and second ratings was -0.11.

The correlation between HSD-s by two separate persons of the same dog was acceptable (r = .65, p < 0.001; Fig 2B). The Bland Altman plots and statistics suggest different people rarely rated the same dog more than two points apart on the scale (2%, i.e. 3 out of 120 people rated outside the critical difference of 1.60). The mean difference between the two people rating the same dog was -0.04.

#### Internal consistency of and associations between personality dimensions

cSPS, fearfulness, and neuroticism in dogs and SPS in humans showed good internal consistency with Cronbach’s alpha being 0.90, 0.84, 0.78, and 0.93, respectively. The Cronbach’s alpha values for the English and German responses were identical for cSPS (0.90) and were only 0.01 different for Fearfulness (English: 0.84, German: 0.85) and Neuroticism (English: 0.78, German: 0.79). The reliability of SPS for responses in German (0.82) was slightly lower than in English (0.94). There was a small difference between cSPS in German and English, with English speakers rating HSD-s 0.20 lower than German speakers (F1, 3633 = 51.19, p < 0.01, R2 = 0.01). Since the effect size of language difference was only small, the data were considered as a whole for further analyses. Despite having a sample biased towards female respondents, no differences were found in the scores provided by men and women for HSD-s (permutation t-test: mean difference = 0.10, p = 0.07) although women scored an average of 0.48 higher in HSP-s than men (permutation t-test: p < 0.001). HSD-s was moderately correlated with fearfulness (r (3633) = .38, p < 0.001) and neuroticism (r (3633) = .41, p < 0.001) scores.

#### Influence of demographics on HSD-s

A general linear mixed model including the “demographic factors” and the random effects of breed and country only had a small effect on the outcome variable HSD-s (the marginal pseudo R2 was 0.04 and the conditional R2 was 0.09). Sheepdogs had the highest HSD-s, followed by Pointing dogs, Scenthounds, Companion dogs, the Pinshers and Schnauzer group, Terriers and Retrievers. The dogs in Germany showed higher HSD-s and those in the UK lower HSD-s than in other countries.

The best-fitting model included the following fixed effects: whether the dog had a previous owner or not (dogs who always had the same owner had a marginally higher HSD-s by 0.29; F_2,3567_ = 26.16, p < 0.001), the country of origin (if this was different from the country of residence, dogs had a higher HSD-s by 0.08; F_1,3539_ = 3.80, p = 0.05), number of people in the household (F_1,3563_ = 0.18, p = 0.67), dog sex (neutered males had higher HSD-s by 0.11 and neutered females by 0.14 compared to intact males; F_3,3568_ = 26.16, p = 0.006), weight (for each kg increase in weight, the HSD-s was decreased by 0.009; F_1,1627_ = 41.98, p < 0.001), and age (for each increase in age, the HSD-s rating was lower by 0.02; F_1,3567_ = 15.12, p < 0.001), and dog’s age at adoption (the older a dog was at adoption, the lower the HSD-s by 0.002; F_1,3563_ = 5.89, p = 0.02). No interactions between the fixed effects were found.

#### Influence of human factors on HSD-s

A general linear mixed model including the “human factors” and the random effects of breed and country only had small effects on the HSD-s (the marginal pseudo R2 was 0.03 and the conditional R2 was 0.07). The best-fitting model included the following fixed effects: the owner’s profession (people belonging to no specified professional category rated dogs as 0.17 higher in HSD-s than did behavior vets and/or trainers; F4,2738 = 3.84, p = 0.004), the interaction between stimulation in the current environment and stimulation in the first environment (if current stimulation was less than first, the HSD-s was rated to be lower by up to 0.80, F16,2734 = 2.32, p = 0.002), communication style (owners that used only positive reinforcement scored their animals 0.93 higher than those who also used strong positive punishment, and 0.55 higher than those who used mild positive punishment), and active time spent with the dog per day (F3,2738 = 1.22, p = 0.29). In a separate linear model, the HSD-s was found to be higher by 0.25 for each increase by 1 in human HSP-s; this accounted for 0.10 of variance based on the R2, and the correlation between the two scores was only moderate (r (3633) = 0.32, p < 0.001).

#### Identification of c-SPS subtraits

The c-SPS questions could be identified into three separate sub-groupings of questions using a PCA (see Table 3).

**Table 3:** Loadings of c-SPS questions into three components based on results of a PCA.

Nr	Subtrait 1 Arousability–ease of excitation	PC1	PC2	PC3
1	My dog is easily stressed, is easily overwhelmed by situations	0.81	0.15	0.06
6	My dog gets nervous quickly or is often nervous	0.77	0.24	0.03
27R	My dog is generally relaxed, can cope well with stress	0.74	0.16	-0.13
11R	My dog is emotionally stable, i.e. he is mostly even-tempered	0.74	0.09	-0.07
9	My dog tends to be uncertain and/or careful	0.72	-0.02	0.26
3	My dog startles easily	0.69	0.06	0.19
17	My dog has problems adapting to changes in every day life and/or bigger changes in life	0.6	0.12	0.12
12	My dog has a tendency to be mistrustful	0.6	0.05	0.16
15R	My dog easily adapts to new environments and can relax there	0.58	0.03	-0.16
21	My dog has trouble when people touch him and/or when things touch him/her…	0.57	0.07	0.15
36	My dog needs a sense of security	0.54	0.05	0.44
19	It takes a long time for my dog to calm down after an arousing event	0.47	0.27	-0.14
28	My dog has problems when he is left alone outside and I move out of sight	0.37	0.09	0.12
	**Subtrait 2 perception/reactivity–low sensory threshold**			
22	My dog is reactive, i.e. he quickly perceives small stimuli and reats quickly and/or strongly to them	0.38	0.57	0.11
26	My dog is always on the alert	0.14	0.69	0.19
25	My dog observes everything that is happening around him	-0.1	0.63	0.45
7	My dog seems to absorb everything that is happening around him/her	0.04	0.59	0.29
38	My dog is easily excitable be it through positive or negative stimuli	0.37	0.58	-0.13
40	My dog reacts strongly to visual stimuli	0.11	0.57	0.22
16	My dog has a subtle perception, i.e. he notices a lot or almost everything	-0.03	0.55	0.53
33	My dog is demanding	0.11	0.47	-0.03
14	My dog tends to be restless	0.4	0.51	-0.16
	**Subtrait 3 emotionality / aesthetic sensitivity**			
32	My dog is sensitive	0.3	0.07	0.7
18	My dog reacts to small changes in voice, i.e. changes in intonation and volume	0.11	0.16	0.61
29	My dog reacts strongly to punishment	0.27	-0.06	0.58
34	My dog is emotional, i.e. reacts strongly to positive and/or negative events	0.36	0.31	0.51
8	My dog reacts when we argue at home	0.23	0.12	0.51
23	My dog is attentive	-0.16	0.44	0.49
2	My dog notices small changes	0.17	0.38	0.48
24	My dog seems thoughtful	-0.07	0.14	0.43
39	My dog is intelligent	-0.14	0.38	0.41
27	My dog is biddable	0.1	0.16	-0.44

PCA analysis led to grouping of the questions into three subtraits, with 13 questions loading highest on Subtrait 1, nine questions on Subtrait 2, and ten on Subtrait 3.

## Discussion

This study is the first to investigate the personality dimension “sensory processing sensitivity” (SPS) in dogs. In a two-step procedure, a questionnaire (referred to as the “HSD questionnaire”) was first developed in a pilot study to identify a personality dimension proposed to be similar to SPS in humans, referred to as “canine SPS” (cSPS), and then validated through an international online survey involving a large sample of dog owners. Results showed that the questions forming the HSD score (HSD-s) had good internal consistency and high intra- and inter-rater reliability, and that the HSD-s was only moderately correlated with the scores for fearfulness and neuroticism. This indicates that the HSD questionnaire has good construct validity and that the HSD-s is at least partly independent from the other personality dimensions included, i.e. neuroticism and fearfulness, similarly to the HSP-s [88] in the human literature. Furthermore, the HSD-s was largely independent of demographic and human factors, including the owner’s level of SPS, and showed consistency across a six-month time period suggesting that the HSD-s reflects a personality dimension, rather than a transient expression of behavior. Principal component analysis lead to the identification of three subtraits, which are comparable to those described in humans.

The use of questionnaires to measure a behavior or personality dimension has several advantages, such as allowing to reach a large population, providing a measure that summarizes the behavioral expression and/or personality over a period of time, and being practical, since the questionnaire can be completed within a short time. Furthermore, the evaluation of personality by proxy and in the form of questionnaires is a commonly used and recognized method with individuals that do not communicate in spoken or signed language, such as human infants or other animal species, including dogs [48,77,79,80,94–96]. Questionnaires have been developed to assess a broad spectrum of behaviors, affective states, and personality dimensions in dogs, such as quality of life in relation to physical illness in general [97], pain [98], atopic dermatitis [99,100] or cardiac disease in particular [101], specific behavior problems, such as canine anxiety [102], canine attention deficit hyperactivity disorder [83], impulsivity [79] or behavioral problems in general [103], and canine personality or temperament [37,77,80,85]. Since a questionnaire measuring SPS had already been validated in humans [57], the same approach to validation was used in dogs.

As the personality dimension SPS had not been described as such in animals, the initial steps of this study relied on the inductive approach chosen by Aron and Aron [57] in humans, which was adapted to dogs. This in itself bore the risk of incurring into circular reasoning, as the initial part of the study was based on the subjective evaluation of dogs as being “highly sensitive”, without having a clear definition of this dimension in dogs to refer to. However, the results from the international online survey retrospectively supported the initial hypotheses of the pilot study and converged towards the identification of a separate and consistent personality dimension which was named ‘cSPS’. First, the sets of questions forming the HSD, fearfulness, and neuroticism scores for the dogs, and the HSP score for the owners, had good internal consistency as shown by high Cronbach’s alphas. This indicates that each set of questions represents the respective personality dimensions well, similar to previous findings in humans [57]. Second, cSPS only moderately related to, and was thus at least partly independent from, neuroticism and fearfulness, similar to the moderate association between neuroticism and SPS found in the human literature [88]. With neuroticism being poorly [40,80,104] and fearfulness [7,105–107] more extensively studied in dogs, the distinction of cSPS from fearfulness carries more weight in this species at this point in time. Third, both the owner–defined as the person who spends most of the time with the dog—and another person who knew the dog well answered the questions for the same dog with a relatively high reliability six months after completing the first questionnaire, indicating a) good intra-rater reliability, b) good inter-rater reliability, c) consistency of cSPS over a relatively long time period, and d) moderate independence of the HSD-s from the owner’s personality to the degree that the owner’s personality did not sufficiently explain the variation in the dogs’ HSD scores. Fourth, the variability of the measured outcome variable (HSD-s) was only to a small degree explained by canine, human and demographic factors reported to influence canine behavior in the literature. Overall, the measures of internal and external validity in this study suggest that a dimension similar to SPS in humans can also be measured in dogs.

SPS in humans has been described by four main characteristics: 1. Strong emotional reactions, 2. Deep processing of information, 3. Awareness of environmental stimuli, and 4. being easily overstimulated [58,62]. Using a principal component analysis, Smoleswka and colleagues [88] identified three subtraits of SPS, referred to as “Ease of Excitation” (EOE), “Aesthetic Sensitivity” (AES); and “Low Sensory Threshold” (LST).

Paralleling the findings in humans, this study revealed the presence of three subtraits of c-SPS in dogs. A direct comparison of the two species is difficult to make, as the questionnaires do not involve exactly the same questions, and the human questionnaire partially includes questions that cannot be applied to dogs, e.g. “do you have a complex, rich inner life” or “are you deeply moved by the arts or music?”. Nevertheless, the three subtraits found for the dogs in this study can be linked to those described in humans. EOE in humans includes questions such as “do you startle easily”, “do other people’s moods affect you?”, “do changes in your life shake you up?”, or “do you find it unpleasant to have a lot going on at once?”. These questions primarily focus on being easily mentally overwhelmed by external (e.g. a lot going on at once) or internal (e.g. hunger) stimuli [88], thereby being more prone to being overstimulated [58,62]. Similarly, questions represented in Subtrait 1 of this study were, for example, “my dog is easily stressed, is easily overwhelmed by situations”, “my dog startles easily”, “my dog has problems adapting to changes in every day life and/or bigger changes in life”, and “my dog easily adapts to new environments and can relax there” (whereby the response to this question was reversed for analysis). It can be concluded that, at least for external stimuli, the term “ease of excitation” can be used for this subtrait in dogs as well.

AES in humans includes questions such as “do you seem to be aware of subtleties in your environment”, “are you conscientious”, “do you notice and enjoy delicate or fine scents, tastes, sounds, works of art” or “are you deeply moved by the arts or music?”. This subtrait can be summarized as involving questions referring to aesthetic awareness [88], but also reflect a deeper processing of information and awareness of environmental stimuli [58,62]. In this study, questions like “my dog reacts to small changes in voice, i.e. changes in intonation and volume”, “my dog notices small changes”, “my dog seems thoughtful” and “my dog is attentive” were found to load onto Subtrait 3, indicative of attention to detail and deeper mental processing. As aesthetic awareness and deep processing of information per se are not possible to evaluate in dogs, we suggest reframing this subtrait into “attention to or awareness of subtleties in the environment”.

Lastly, examples for questions found in the LST subtrait in humans are “are you easily overwhelmed by things like bright lights, strong smells, coarse fabrics, or sirens close by?”, “do you make a point to avoid violent movies and TV shows?” or “are you bothered by intense stimuli, like loud noises or chaotic scenes?”. The main contents of this subtrait focusses on being (unpleasantly) aroused by environmental stimulation [88], which can lead to strong emotional reactions [58,62]. Subtrait 2 in this study involved questions that could be related to this, e.g. “my dog is reactive, i.e. he quickly perceives small stimuli and reacts quickly and/or strongly to them”, “my dog is easily excitable be it through positive or negative stimuli”, “my dog reacts strongly to visual stimuli”, or “my dog seems to absorb everything that is happening around him/her”. Therefore, the intensity and rapidity of a response, attention to detail and awareness of what is happening in the environment, allows for a comparison with LST in humans to be drawn.

When using questionnaires to assess an animal’s behavior or characteristics of the animal in certain situations, owners usually have to estimate to which extent such characteristics apply to their pet on a scale. Hence, there are two main aspects that can influence the resulting score: a) the owner’s interpretation of his or her pet’s behavior, and b) the dog’s actual behavior, which in turn can both be influenced by many factors.

With regard to the aspects that may have influenced the participation in the online survey and the interpretation of its questions, it is possible that owners with certain personalities may have been more likely to be interested in studies of dog personality, or that owners who had more background knowledge on canine behavior could interpret their dogs’ behaviors differently from owners with less experience. It has been shown that owners with differing personalities tend to behave differently around their dogs, e.g. owners scoring high on neuroticism and openness tended to give more commands, and owners scoring high on the extraversion scale praised their dogs more, in a study run by Kis and colleagues [108]. Moreover, working dogs belonging to owners with certain personalities performed differently in working tasks [109]. Not only the behaviors of both dog and owner have been found to differ with varying personalities of the owners, but also how the owners perceive their dogs and what they value in them seem to play important roles. Owner satisfaction, for example, was higher when their dog’s or cat’s behavior was similar to their owners’ inter-personal behavior styles [110]. Owners scoring high on neuroticism have also been shown to be more likely to evaluate their dogs to be nervous, anxious, and emotionally less stable, and those scoring high on extraversion to assess their dogs to be energetic, enthusiastic, and sociable[111]. In a large-scale study, Turcsán and colleagues [39] found significant positive correlations between owners and dogs for the (self-evaluated) personality dimensions of neuroticism, extraversion, conscientiousness, agreeableness, and openness. However, these associations (all except for openness) remained significant also when an independent person evaluated the personality of both dog and owner, thus the authors concluded that the dog—owner similarities could not be only due to owner self-projection. Hence it was important to investigate the relationship between the HSP-s and HSD-s in this study. Even though owners with a higher HSP-s evaluated their dogs to be slightly higher on the canine sensitivity scale, the HSP-s only had a small effect on the HSD-s. Additionally, the inter-rater reliability was good, further supporting the indication that the owner’s level of sensitivity did not largely affect the scoring of their dogs in this questionnaire. Only the correlation between the dog’s and the primary owner’s personalities was analyzed in this study. However, the social environment of the dog was most likely more complex, due to the possible interactions with other family members and/or animals, and these factors could have potentially affected the personality development of the dog. As the association between primary owner’s HSP-s and dog’s HSD-s was only moderate, it could be hypothesized that the personality of a secondary person less close to the dog would have even less of an influence. However, this remains to be systematically assessed in future studies.

Another factor which could have potentially affected how dogs were perceived by the owners is the owners’ cultural background. Pet owners in the Bahamas, for example, interacted with and evaluated their pets differently from pet owners in the United States of America, independent of animal species [111], and owners of German shepherd dogs in the United States evaluated their dogs to be more confident and aggressive than Hungarian owners of the same breed [112]. These previous studies may offer an explanation for the slight differences that were found in the HSD-s of dogs evaluated by people living in the UK, Germany and Canada. The effect of country on the HSD-s in the final model, however, was only small. Moreover, the amount of experience participants had with their dog or with dogs in general, or the owner’s sex and age could have influenced the interpretation and understanding of canine behavior [91]. In this study however, the owner’s profession and sex were found to have again a small effect on the HSD-s.

The psychological development and the behavior of individuals have been proposed to be influenced by a large array of factors in different species. These factors range from breed [113,114] and personality traits in dogs [115], to the evidence in various species of pre- and perinatal influences of hormones, e.g. androgens in humans [116,117] or stress hormone levels of the mother [118,119], on the behavioral development of the young. Likewise external factors after birth, such as quality of maternal care [120,121], degree, variety and quality of stimulation during the first months of life [107,122,123] and later on in life in dogs [124] have been described to affect behavior. Characteristics of the owner, e.g. type of communication [125–127] or experience with dogs [91,128,129], must be taken into consideration as well. Lastly, the dog’s health can also affect how a dog behaves [130–132]. Those factors most frequently reported in the literature were included in this study. Based on the history of breeding dogs for particular uses, it might be expected that certain aspects of behavior would have a greater or lesser likelihood to occur in specific breeds. However, the information in the literature regarding the effect of dog breed on personality and behavior are contradictory. The extent and direction of these effects seem to depend on which dimensions the studies focused on. Asp and colleagues [133], for example, looked at several behavior clusters across 20 breeds grouped into working and non-working dogs and found a variation in the behaviors “trainability”, “aggression”, and “fear”. Whereas Mirkó and colleagues [37] concluded that pet dog breed-groups (according to the FCI Groups) and breeds differed only slightly in terms of the personality dimensions studied, i.e. stranger-directed sociability, aggressiveness, activity, and trainability. Recent research suggests that within-breed differences (partially based on training experience) might be more significant than between breed differences [115,134,135]. The conscious choice of including all available dog breeds in this study was based on the above-mentioned inconsistencies in the literature, on the growing research focus on individuals as opposed to groups [115], and on data from cross-cultural research in humans showing that the prevalence of highly sensitive individuals seems to be very similar in different human cultures independent of race [60]. Therefore, if there was a genetic component to cSPS in the dog, then it would be expected to be found within every population and every sub-population, i.e. also within every breed. Hence, in this study, one aim was to determine whether cSPS could be measured in the species “*canis familiaris*”, not in any specific breed. Although there were slight differences in the means of the separate breed groups, our results support the independence of cSPS from dog breed. However, more research on the distribution of the dimension within specific breeds is needed to further confirm our finding.

An animal’s behavior can be largely affected by sex, including neutering status in species to which this applies. In humans, women score higher on certain personality traits, e.g. neuroticism, anxiety, and nurturance [136] while men do on others, e.g. self-esteem, hostility, and assertiveness [136]. However, differences between sex appear to be much smaller than inter-individual differences [136]. Our results reflect this finding in dogs, with neutered individuals of both sexes scoring marginally higher on the cSPS scale than intact male dogs. SPS in humans is reported in both men and women, with women generally scoring higher on the scale [57,67]. One possible explanation for this finding might be the fact that in Western culture, sensitivity is regarded as something negative, particularly in men, which might influence how men want to be perceived and, hence, how they choose to fill in a questionnaire [57]. This cultural bias is not as likely to exist in dogs. Sex, including neutering status, involves sex hormones, of which testosterone and estrogen have been shown to have a protective effect on the development of fear in species such as the rat [137,138], mouse [139] and sheep [140,141]. Male dogs and intact dogs were found to be bolder than female and neutered dogs, respectively [18]. Thus, neutered individuals might show slightly higher fearfulness or reactivity compared to intact ones [142], due to the lower levels of gonadal steroids, and hence score slightly higher on those questions overlapping with fearfulness in the HSD questionnaire. This might be a further possible explanation for the differences found in scores of neutered and intact male dogs.

An important aspect that must be addressed is whether the measured HSD-s is not actually measuring behavior problems. Although the model including the human factors as a whole only explained little variance in the HSD-s, the owner’s communication style and the interaction between stimulation in the current environment and stimulation in the first environment had a relatively large effect size on the HSD-score. The aspect of owner-dog interaction considered in this study was represented by the type of communication owners tended to use based on self-reporting, i.e. positive punishment, negative punishment, and positive reinforcement. Positive punishment has been shown to be associated with an increased incidence of behavior problems in several studies [126,127,143]. The fact that owners who used only positive reinforcement tended to score their dogs higher on the HSD-scale than did owners who used positive punishment (i.e., dogs who were punished had lower HSD-scores than dogs who were not), indicates that the HSD-questionnaire is actually measuring something different from behavior problems. Moreover, dogs whose current environment was less stimulating than the first one were scored lower on the HSD-scale. The mismatch between the degree of stimulation in the current (more stimulating) and first (less stimulating) environment has been considered a risk factor for development of behavior problems in dogs, primarily fearful behavior and anxiety [107,144–146]. If the HSD-s were coinciding with behavior problem, a higher HSD-s would be expected in dogs whose first environment was less stimulating than the current, however this was not the case in this study. Hence, this could be interpreted as a further indication that the HSD questionnaire is not measuring behavior problems.

## Conclusion

In conclusion, our HSD questionnaire fulfilled the requirements of internal consistency, reliability and (partial) independence of the HSD-s from the personality dimensions neuroticism and fearfulness, indicating that c-SPS is a measurable personality dimension in dogs. Furthermore, the overall small influence of demographic and human factors on the HSD-s suggests good generalizability of our findings on c-SPS to the dog population, possibly due to the presence of an underlying genetic basis for this dimension within the dog species. The c-SPS trait in dogs seems to consist of three subtraits similar to the ones described in humans: Aesthetic Sensitivity, Ease of Excitation, and Low Sensory Threshold.

The hypothesis has been made in the human literature that SPS might be a dimension underlying other personality dimensions involving a higher vulnerability to stress and negative life events, such as neuroticism, negative emotionality, vulnerability to depression, or inhibitedness [58]. Similarly, the argument could be made that in animals, too, these observed modes of reaction might be due to the interaction of a possible underlying predisposition, such as cSPS, with other personality dimensions.

The identification of a personality dimension that in humans is hypothesized to underlie already studied dichotomous strategies in response to novel or stressful situations has the potential of being of great practical interest and importance in the field of clinical veterinary behavior and for animal welfare in general. Further work is needed to confirm the internal and external validity of HSD-s, and to investigate the relationship between c-SPS and vulnerability to environmental factors.

## Supporting information

S1 Appendix Original 112 questions of the pilot study with sources. (PDF)

S2 Appendix The highly sensitive dog questionnaire (HSD-Q). (PDF)

S3 Appendix Online survey general questions. (PDF)

S1 Dataset Dataset. (XLS)
