# Supplementary material for: Development of the “Highly Sensitive Dog” questionnaire to evaluate the personality dimension “Sensory Processing Sensitivity” in dogs
Source: PLoS One. 2017 May 16;12(5):e0177616. doi: 10.1371/journal.pone.0177616 (PMC5433715; doi:10.1371/journal.pone.0177616)
Supplement: S1 Appendix — (PDF) [file pone.0177616.s001.pdf]

S1 Appendix - The original 112 questions of the pilot study with sources

	Questions German	English translation of questions	Source
1	Mein Hund ist anhänglich, d.h. er ist generell dort, wo ich auch bin.	My dog is clingy, he usually is where I am.	Interview
2	Mein Hund ist aufdringlich.	My dog is pushy.	Interview
3	Mein Hund beobachtet alles, was um ihn herum geschieht.	My dog observes everything that is happening around him.	Interview, PANAS (5R)
4	Mein Hund braucht viel Ruhe / Erholungszeit.	My dog needs a lot of peace and quiet, time to recover.	HSP-Q (5)
5	Mein Hund braucht körperliche Nähe.	My dog needs physical proximity.	Interview
6	Mein Hund braucht Sicherheit.	My dog needs a sense of security.	Interview
7	Mein Hund ist draufgängerisch, mutig.	My dog is ballsy, courageous.	Interview
8	Mein Hund ist emotional, reagiert auf positive und/oder negative Ereignisse stark.	My dog is emotional, i.e. reacts strongly to positive and/or negative events.	Interview, HSC-Q (23), C-BFI (24R)
R9	Mein Hund ist eher extrovertiert.	My dog is rather extroverted.	Interview
10	Mein Hund ist eher introvertiert.	My dog is rather introverted.	Interview
11	Mein Hund ist fordernd.	My dog is demanding	Interview, HSC-Q (13)
12	Mein Hund ist frech.	my dog is cheeky	Interview
R13	Mein Hund ist eher geduldig.	My dog is rather patient.	I-Q (17R)
14	Mein Hund ist schnell "hyperig".	My dog is "hyper" quickly.	Interview, Monash (E)
15	Mein Hund reagiert impulsiv.	My dog reacts impulsively	Impulsivity-Q (3)
16	Mein Hund beobachtet und wartet im Allgemeinen, bevor er handelt.	my dog general observes and watches, before he acts.	HSC-Q (21), I-Q (7R)
17	Mein Hund ist interessiert.	My dog is interested	HSC-Q
18	Mein Hund ist liebesbedürftig.	My dog is needy of love.	Interview
19	Mein Hund ist tendenziell misstrauisch	My dog has a tendency to be mistrustful	Interview, C-BFI (22R)
20	Mein Hund reagiert darauf, wenn wir zuhause streiten.	My dog reacts when we argue at home.	Interview, HSC-Q (15), HSP-Q (18, 25), C-BFI (34R)
21	Mein Hund ist reaktiv, d.h. er nimmt geringe Reize generell schnell wahr und reagiert schnell und/oder stark darauf	My dog is reactive, i.e. he quickly perceives small stimuli and reacts quickly and/or strongly to them.	Interview, I-Q (16)
22	Mein Hund ist schreckhaft.	My dog startles easily	HSC-Q (1), HSP-Q (13), PANAS (3)
23	Mein Hund ist sensibel.	My dog is sensitive	HSP-Q (27), Monas (N)
24	Mein Hund ist verschmust.	my dog is cuddly	Interview
25	Mein Hund hat "die Antennen immer ausgefahren"	My dog always "has his antennae up", is always on the alert	Interview
26	Mein Hund hat eine feine Wahrnehmung, nimmt viel oder fast alles wahr.	My dog has a subtle perception, i.e. he notices a lot or almost everything.	Interview, HSC-Q (5), HSP-Q (15)
27	Mein Hund kommuniziert auf eine feine Art und Weise mit mir, anderen Personen und/oder Hunden.	my dog communicates in a gentle way with me, other people and/or other dogs.	Interview
28	Mein Hund scheint alles, was um ihn herum läuft, aufzusaugen.	My dog seems to absorb everything that is happening around him.	Interview
29	Mein Hund übernimmt die Emotionen anderer, reagiert auf meine Stimmung und/oder die Stimmung anderer.	My dog takes on my emotions, reacts to my mood or that of others.	Interview, HSC-Q (15), HSP-Q (3), C-BFI (37)
30	Mein Hund reagiert stark auf Bestrafung	My dog reacts strongly to punishment.	HSC-Q (4)
31	Mein Hund reagiert schon auf geringe Veränderungen der Stimme / Intonation / Lautstärke der Stimme.	My dog reacts to small changes in voice, i.e. changes in intonation and volume.	Interview
32	Mein Hund braucht viel Zeit, bis er zu jemandem Vertrauen fasst.	My dog takes his time to trust somebody.	HSC-Q (21), C-BFI (22R)
33	Mein Hund verträgt Hitze schlecht.	My dog has trouble tolerating heat.	Interview
34	Mein Hund verträgt Kälte schlecht.	My dog has trouble tolearting cold.	Interview
35	Mein Hund hat Mühe mit Veränderungen im Alltag, im Tagesablauf und/oder Lebensveränderungen (z.B. veränderte Routine, Besuch, Partnerwechsel, Ferien, Umzug, etc.)	My dog has problems adapting to changes in every day life (e.g. changes in routine, visitors, chnage of partner, vacation, moving, etc.)	HSC-Q (11), HSP-Q (21), PANAS (15R,21)
36	Mein Hund braucht lange, bis er sich nach einer Erregung erholt und wieder "oben runter" kommt.	It takes a long time for my dog to calm down after an arousing event.	HSC-Q (10), HSP-Q (11), PANAS (6), I-Q (13)
R37	Mein Hund hat eine gute Konzentrationsfähigkeit.	My dog 's a good power of concentration.	Interview, HSC-Q (22R), HSP-Q (26), C-BFI 42R), I-Q (12)
R38	Mein Hund kann sich lange konzentrieren.	My dog has a good concentration span.	Interview, C-BFI (42R), I-Q (12)
R39	Mein Hund kann sich schnell an eine neue Umgebung gewöhnen und sich dort entspannen.	My dog easily adapts to a new environment and can relax there	PANAS (10), I-Q (11R)
40a	Mein Hund ist leicht gestresst, ist schnell mit Situationen überfordert	My dog is easily stressed , easily overwhelmed by situations.	Interview, HSC-Q (19), HSP-Q (1,7,9,14,16,19,23,25), C-BFI (9R)
41	Mein Hund initiiert häufig Spiele mit anderen Hunden und/oder Menschen.	My dog often initiates play with other dogs or people	Interview
42	Mein Hund hat eine sanfte Art des Umgangs / der Kommunikation / des Spielens mit anderen Hunden und/oder Menschen.	My dog has a gentle way of communicating/playing with other dogs and / or people.	Interview, HSC-Q (16)
R43	Mein Hund entspannt sich schnell im Auto und kann dort auch schlafen.	My dog relaxed quickly in the car and can sleep.	Interview
44	Mein Hund ist in Menschenmengen eher angespannt, nervös, zeigt Anzeichen von Stress.	My dog is rather tense, nervous and shows signs of stress in crowds	PANAS (10)
45	Mein Hund ist eher schmerzempfindlich, "es Mimösli".	my dog is sensitive to pain.	HSC-Q (18), HSP-Q (4)
46a	Mein Hund hat Mühe damit, wenn ihn Leute anfassen und/oder wenn ihn etwas berührt (z.B. Gstältli, Mantel, nasse Blätter, etc.).	My dog has trouble when people touch him and/or when things touch him/her (e.g. harness, coat, wet leaves, etc.)	Interview, HSC-Q (2,12), I-Q (4)
47	Mein Hund reagiert stark auf Geräusche.	my dog reacts strongly to sounds.	HSC-Q (19), HSP-Q (7,9,25), PANAS (12)
48	Mein Hund reagiert stark auf visuelle Reize.	my dog reacts strongly to visual stimuli	HSP-Q (7)
49	Mein Hund reagiert stark auf Gerüche.	my dog reacts strongly to smells	HSC-Q (7), HSP-Q (7)

	Questions German	English translation of questions	Source
50	Mein Hund bemerkt kleine Veränderungen.	my dog notices small changes.	HSC-Q (20), HSP-Q (2,22)
51	Mein Hund mag es nicht, manipuliert zu werden (z.B. beim Tierarzt oder wenn ich eine Zecken entfernen oder Ohrentropfen verabreichen muss)	my dog does not like to be manipulated (e.g. at the vet's or when I need to remove a tic or give him ear drops)	Interview, I-Q (4)
52	Mein Hund hat Mühe damit, wenn ich ihn draussen warten lasse und mich ausser Sichtweite begebe.	My dog has problems when he is left alone outside and I move out of sight.	Interview
53	Mein Hund hilft es, wenn ich ihm in für ihn schwierigen Situationen sage, was er tun soll, anstatt die Situation "selbst in die Hand zu nehmen".	It helps my dog when I tell him what to do in difficult situations instead of "taking on the situation" himself.	Interview
54	Mein Hund bleibt erstmals auf Distanz und beobachtet, wenn er mit etwas Neuem konfrontiert wird.	My dog initially stays at a distance and observes when he is confronted with something new.	HSC-Q (21), I-Q (7R)
55	Mein Hund ist aggressiv.	My dog is aggressive	Monash (A)/R, I-Q (5,9)
56	Mein Hund ist ängstlich.	my dog is fearful	Monash (N), PANAS (1R)
57	Mein Hund ist aktiv, braucht Bewegung, hat viel Energie, ist lebhaft.	my dog is active, needs a lot of exercise, has a lot of energy, is lively.	Monash (E), PANAS (11,20), C-BFI (11)
58	Mein Hund ist aufmerksam	my dog is attentive	Monash (T), PANAS (5R)
59	Mein Hund ist dominant	my dog is dominant	Monash (M)
60	Mein Hund ist begierig, willig.	my dog is eager, willing.	Monash (E)
61	Mein Hund ist leicht erregbar, "fährt schnell hoch", sei es durch positive oder negative Reize.	My dog is easily excitable be it through positive or negative stimuli.	Monash (E), PANAS (7), I-Q (18)
62	Mein Hund ist freundlich, lieb.	my dog is friendly, nice	Monash (A), C-BFI (32)
63	Mein Hund ist fröhlich, lustig, sorglos, unbekümmert, immer gut drauf.	my dog is happy, funny, carefree, happy-go-lucky, always in a good mood.	Monash (A)
R64	Mein Hund ist fügsam.	my dog is biddable.	Monash (A)
65	Mein Hund gehorcht im Allgemeinen gut.	my dog generally obeys well.	Monash (T)
66	Mein Hund ist eher hyperaktiv.	my dog is rather hyper active	Monash (E)
67	Mein Hund ist intelligent	My dog is intelligent	HSC-Q (6,17), Monash (T)
68	Mein Hund ist neugierig, muss seine Nase überall hineinstecken.	my dog is curious, always has to poke his nose into everything.	Monash (M), C-BFI (10), I-Q (15)
69	Mein Hund hat eine sanfte Natur.	my dog is of a gentle nature.	Monash (A)
R70	Mein Hund ist selbstbewusst, selbständig, unabhängig.	my dog is confident, independent	Monasch (M)
71	Mein Hund ist gut trainierbar.	my dog is easily trainable	Monash (T), I-Q(10)
72	Mein Hund ist häufig überschwänglich.	my dog is often exuberant	Monash (E)
73	Mein Hund ist eher unruhig.	My dog tends to be restless.	HSP-Q (11), Monash (E), PANAS (13R), C-BFI (9R, 21R)
74	Mein Hund ist ein Opportunist, er nützt Situationen gerne zu seinem Vorteil aus.	my dog is an opportunist, he takes advante of situations.	Monash (M)
75a	Mein Hund ist eher unsicher und/oder vorsichtig.	My dog tends to be uncertain and/or careful.	Monash (N), C-BFI (8R)
76	Mein Hund ist eher unterwürfig.	my dog is submissive	Monash (N)
77	Mein Hund ist tendenziell ruhig	my dog tends to be calm	Monash (E)
78	Mein Hund zeigt grossen Enthusiasmus, ist begeisterungsfähig, leidenschaftlich.	my dog shows great enthusiasm, passionate	Monash (E), C-BFI (16)
79	Mein Hund hat eine durchsetzungsfähige Persönlichkeit, ist entschlossen, eigenwillig, stur, hat einen starken Willen.	my dog has an assertive personality, is decisive, stubborn, headstrong, has a strong will.	Monash (M), PANAS (4,15), C-BFI (26,28,38), I-Q (8)
R80	Mein Hund ist generell entspannt und kann gut mit Stress umgehen.	My dog is generally relaxed and copes well with stress	Monash (A), PANAS (13), C-BFI (9)
81	Mein Hund ist kontaktfreudig, gesellig.	my dog is sociable	Monash (A), C-BFI (36)
82	Mein Hund macht Dinge gründlich.	my dog is conscientious.	HSC-Q (14), HSP-Q (12), Monash (M), C-BFI (34R)
83	Mein Hund ist eher schüchtern, gehemmt, zurückhaltend.	My dog is rather shy, inhibited, cautious	HSP-Q (27), Monash (N), C-BFI (31)
84	Mein Hund ist sensibel auf die Bedürfnisse und Gefühle anderer.	My dog is sensitive to needs and feelings of others.	HSC-Q (15), C-BFI (37
85	Mein Hund wird schnell nervös oder ist häufig nervös.	My dog gets nervous quickly or is often nervous.	Monash (N), C-BFI (39)
86	Mein Hund ist verlässlich, ich kann mich auf ihn/sie verlassen.	my dog is reliable, I can rely on him.	Monash (T), C-BFI (13)
87	Mein Hund ist kann angespannt sein.	my dog can be tense.	C-BFI (14)
88	Mein Hund ist ist ausdauernd, hartnäckig, beharrlich, hält durch, bis die Arbeit fertig ist.	my dog is persistent, insistent, perseverant, keeps on going until the work is done.	Monash (M)
R89	Mein Hund ist bleibt ruhig in angespannten, lauten Situationen oder Situationen, in denen viel läuft.	My dog stays calm in tense, loud situations or situations where a lot is going on.	HSC-Q (19), HSP-Q (5,19), PANAS (10, C-BFI (34)
90	Mein Hund ist scheint häufig "down" oder deprimiert.	My dog often seems to be "down" or depressed.	C-BFI (4)
91	Mein Hund ist kann kühl und distanziert sein.	My dog is aloof and distant.	C-BFI (27)
92	Mein Hund ist ist ein Einzelgänger, eher distanziert, reserviert.	My dog is a loner, rather distant and reserved.	C-BFI (6)
R93	Mein Hund ist ist emotional stabil, d.h. meistens ausgeglichen und nicht schnell aus der Fassung zu bringen.	My dog is emotionally stable, i.e. he is mostly even-tempered and not easily unnerved.	C-BFI (24)
R94	Mein Hund ist ist einfallslos.	my dog is unimaginative.	C-BFI (20)
95	Mein Hund ist ist erfinderisch, kreativ, findet neue Wege, das zu erhalten, was er möchte, lässt sich neue Wege einfallen, Dinge zu machen.	my dog is creative, inventive, finds new ways to get what he wants or to do things.	C-BFI (5)
96	Mein Hund ist ist tendenziell faul, macht nicht mehr als er muss.	my dog tends to be lazy, does not do more than he has to.	PANAS (14), C-BFI (23)
97	Mein Hund ist macht generell mit, wenn ich etwas von ihm will, er ist kooperativ, gibt viel.	my dog generally participates when I want something from him, he is cooperative, and gives a lot.	C-BFI (41)
98	Mein Hund ist kann launisch sein.	my dog can be moody.	C-BFI (29)
99	Mein Hund ist ist leicht durch Dinge abgelenkt.	My dog is easily distracted.	C-BFI (42)
100	Mein Hund ist ist lernwillig, lernt und macht gerne neue Dinge.	my dog is eager to learn, likes to learn and do new things.	C-BFI (35)
101	Mein Hund ist scheint sich viele Sorgen zu machen.	My dog seems to worry a lot.	C-BFI (19)
102	Mein Hund ist scheint nachdenklich	My dog seems thoughtful.	C-BFI (15)
103	Mein Hund ist wird schnell nervös oder ist häufig nervös.	My dog is nervous quickly or often.	C-BFI (39)
R104	Mein Hund ist vertraut in der Regel schnell.	My dog tends to trust quickly.	C-BFI (22)
R105	Mein Hund ist hat selten Angst	My dog is rarely frightened	Monash (NR), PANAS (1)

	Questions German	English translation of questions	Source
106	Mein Hund ist wird sehr aufgeregt, wenn wir uns auf einen Spaziergang vorbereiten (z.B. wenn er die Leine sieht oder das Wort "Spaziergang" hört, etc.)	My dog gets very excited when we get ready for a walk (e.g. when he sees the lead or hears the word "walk", etc.)	PANAS (2)
107	Mein Hund ist hat eine bestimmte Angst oder Phobie.	My dog has a specific fear or phobia.	PANAS (8)
108	Mein Hund ist versucht, aus dem Garten auszubrechen.	My dog tries to escape from the yard.	PANAS (9)
109	Mein Hund ist hat Angst vor Geräuschen im Fernsehen oder Radio.	My dog is afraid of noises in the tv or radio.	PANAS (12)
110	Mein Hund ist hat Angst vor dem Staubsauger oder anderen alltäglichen Haushaltsgeräten.	my dog is afraid of the vacuum cleaner or other everyday household devices.	PANAS (16)
111	Mein Hund ist muss stark motiviert werden, um bei energieaufwändigen Aktivitäten mitzumachen.	My dog has to be strongly motivated to do something energy-consuming.	PANAS (17)
112	Mein Hund ist bleibt frech, auch wenn ich ihn mehrfach zurechtweise.	My dog keeps on being cheeky even after being reprimanded repeatedly.	PANAS (18)

HSC-Q = Highly Sensitive Child Questionnaire [85], HSP-Q = Highly Sensitive Person Questionnaire [57], C-BFI = Canine Big Five Inventory [86],
PANAS = Positive and Negative Activation Score [79], I-Q = Impulsivity Questionnaire [77]
(number) = Question number in original questionnaire
Monash Questonnaire [78]: E = extraversion, M = motivation/self-assuredness, N = neuroticism, T = training focus, A = amicability

grey = Questions with significantly different mean scores between HSD and nHSD group
R = Questions with reversed scores
