# Supplementary material for: Development of the “Highly Sensitive Dog” questionnaire to evaluate the personality dimension “Sensory Processing Sensitivity” in dogs
Source: PLoS One. 2017 May 16;12(5):e0177616. doi: 10.1371/journal.pone.0177616 (PMC5433715; doi:10.1371/journal.pone.0177616)
Supplement: S2 Appendix — (PDF) [file pone.0177616.s002.pdf]

S2 Appendix - The Highly Sensitive Dog Questionnaire with original sources of questions

(the numbers under each source refer to question numbers in the corresponding original questionnaire)

Number	Question	Neuroticism (N) Fearfulness (F)	HSC [85]	HSP [57]	Interview	Monash [78]	C-BFI [86]	PANAS [79]	Impulsivity [77]	Guide dogs [34]
1	My dog is easily stressed, is easily overwhelmed by situations.		19	1,7,9,14,16,19,23,25	x		9R			
2	My dog notices small changes.		20	2,22						
3	My dog startles easily	N	1	13				3		
4	My dog is fearful	F						1R		
5	My dog is obviously startled by loud or unexpected sounds.	F								x
6	My dog gets nervous quickly or is often nervous.	N				N	39			
7	My dog seems to absorb everything that is happening around him/her.				x					
8	My dog reacts when we argue at home.		15	18,25	x		34R			
9	My dog tends to be uncertain and/or careful.	N				N	8R			
10	my dog is submissive	N				N				
11R	My dog is emotionally stable, i.e. he is mostly even-temepered and not easily unnerved.						24			
12	My dog has a tendency to be mistrustful				x		22R			
13	My dog is obviously startled by odd or unexpected things or objects.	F								x
14	My dog tends to be restless.			11		E	9R,21R	13R		
15R	My dog easily adapts to a new environmenst and can relax there							10	11R	
16	My dog has a subtle perception, i.e. he notices a lot or almost everything.		5	15	x					
17	My dog has problems adapting to changes in every day life (e.g. changes in routine, visitors, etc) and/or bigger changes in life, e.g. change of partner, rearranging furniture, going on holidays, moving home, etc.)		11	21				15R,21		
18	My dog reacts to small changes in voice, i.e. changes in intonation and volume.				x					
19	It takes a long time for my dog to calm down after an arousing event.		10	11				6	13	
20	My dog is anxious or uneasy in new situations.	F								x
21	My dog has trouble when people touch him and/or when things touch him/her (e.g. harness, coat, wet leaves, etc.)		2,12		x				4	
22	My dog is reactive, i.e. he quickly perceives small stimuli and reacts quickly and/or strongly to them.				x				16	
23	My dog is attentive.					T		5R		
24	My dog seems thoughtful.						15			
25	My dog observes everything that is happening around him.				x			5R		
26	My dog is always on the alert				x					
27R	My dog is generally relaxed, can cope well with stress.					A	9	13		
28	My dog has problems when he is left alone outside and I move out of sight.				x					
29	My dog reacts strongly to punishment.		4							
30	My dog is very impulsive, i.e. s/he has sudden, strong urges to act, acts without forethought, is impatient.	*							3	
31R	my dog is biddable.					A				
32	My dog is sensitive	N		27		N				
33	My dog is demanding		13		x					
34	My dog is emotional, i.e. reacts strongly to positive and/or negative events.		23		x		24R			
35	My dog is timid.	*	27			N	31			
36	My dog needs a sense of security.				x					
37	My dog backs away from or is reluctant to pass objects no the street (such as collecting boxes, bin bags or children's ride-on toys).	F								x
38	My dog is easily excitable be it through positive or negative stimuli.					E		7	18	
39	My dog is intelligent		6,17			T				
40	My dog reacts strongly to visual stimuli.		7							
41R	My dog is rarely frightened	F, N				NR		1		
42	My dog has a specific fear or phobia	F						8		

grey shading = questions added to HSD questionnaire, which were either not significantly different or not included in the pilot study, numbers refer to question in corresponding questionaire

Monash Questonnaire [78]: E = extraversion, M = motivation/self-assuredness, N = neuroticism, T = training focus, A = amicability

* questions originally added to the questionnaire, but not analyzed

R = questions with reversed scoring
