# Supplementary material for: Development of the “Highly Sensitive Dog” questionnaire to evaluate the personality dimension “Sensory Processing Sensitivity” in dogs
Source: PLoS One. 2017 May 16;12(5):e0177616. doi: 10.1371/journal.pone.0177616 (PMC5433715; doi:10.1371/journal.pone.0177616)
Supplement: S3 Appendix — (PDF) [file pone.0177616.s003.pdf]

S3 Appendix - Online survey general questions

Question	Response options
**Owner**	
Would you like feedback on the results of the study? If yes, please give us your email address in the comment box below	no<br>yes
In which country do you currently live?	Switzerland<br>Germany<br>Austria<br>UK<br>USA<br>Canada
What is your age?	< 18 years<br>18-30 years<br>31 - 65 years<br>> 65 years
What is your gender?	male<br>female
Do you belong to any of the following professional / academic categories?	dog trainer<br>dog trainer specialised in behaviour<br>veterinarian<br>behaviour veterinarian<br>university student<br>university employee<br>none of the above
How did you hear about this study?	Flyer<br>Email<br>Other (with comment option)
**Dog**	
What is your dog's name?	
How old is your dog at this point in time?	years and months
How old was your dog when you got him/her?<br>If you don't know exactly, please provide your best estimate.	years and months
What breed is your dog?<br>If it s/he is a mixed breed, please indicate "mixed breed"	
What is your dog's gender?	male, intact<br>male, sterilised/neutered<br>female, intact<br>female, sterilised/neutered
What is your dog's current weight?	kg or lb
**Communication / Training**	
How do you let your dog know when s/he does something right?	I give him/her food treats (R+)<br>I pet or cuddle him/her (R+)<br>I use a clicker or marker word (R+)<br>I praise him/her with my voice (R+)<br>I play with him/her and/or give him/her a toy (R+)<br>I work with a prey dummy (R+)<br>I give him/her friendly attention (R+)<br>I reward my dog with other things s/he enjoys, e.g. take her for a walk, letting him play, letting her sniff, letting him dig, etc)<br>other<br>none of the above
How do you let your dog know when s/he does something wrong?	I withhold a reward (treats, petting, etc.) (P-)<br>I use my voice, e.g. shout or use a sharp tone of voice (P+)<br>I turn him/her onto his/her back (P+)<br>I give him/her a time-out, e.g. by locking him/her away for a while (e.g. putting him in another room / in his/her crate) (P-)<br>I press my dog to the ground (P+)<br>I do obedience work (P+)<br>I use a spray collar (P+)<br>I ignore him/her (P-)<br>I use a shock collar (P+)<br>I make him/her submit (P+)<br>I tug on the lead (P+)<br>I put my hand over his/her muzzle (P+)<br>I use a choke collar (P+)<br>I use a noise like disks / a bottle or can filled with stones or similar / keys to stop the behavior (P+)<br>I might kick or hit my dog (P+)<br>I spray my dog with water (P+)<br>I tap my dog on the nose (P+)<br>other<br>none of the above

Question	Response options
**Health**	
Does your dog suffer or has s/he in the past suffered from any severe, persistent or recurring physical illnesses?<br>Please briefly decribe the health problem/s of your dog in the comment box below.	yes/no<br><br>if yes, please indicate what category of physical illness it is/was:<br>Digestive tract (e.g. diarrhea, constipation, vomiting, tooth problems, salivation, food intolerance, etc.)<br>Respiratory tract (e.g. coughing, sneezing, secretion out of nostrils, sounds when breathing, etc.)<br>Cardiovascular system (e.g. heart murmur, out of breath quickly, irregular heart beat, etc.)<br>Skin (e.g. infections, itchiness/scratching, hair loss, ear infections, wounds, etc.)<br>Sensory organs (e.g. decreased vision, cataracts, hearling loss, etc.)<br>Urinary tract (e.g. bladder infections, kidney problems, etc.)<br>Reproductive tract (e.g. cryptorchism, pyometra, prostate problems, etc)<br>Immune system (e.g. allergies, auto-immune disease, reaction to vaccinations, frequent inflammations, etc.)<br>Locomotor apparatus (e.g. arthritis, fractures, limping, gait abnormalities, muscle atrophy, trembling, etc.)<br>Nervous nsystem (e.g. seizures / epilepsy, cauda equina, etc.)<br>Metabolic (e.g. diabetes mellitus, hypothyroidism, Chushings, Addison's, etc.)<br>Infections (e.g. bacterial, viral, fungal, tick-borne disease (e.g. borreliosis), leishmaniosis, etc.)<br>Surgeries (e.g. castration/sterilisation, fractures, gastric volvulus, (bite-) wounds, etc.)<br>Other
Does your dog show any behavior problems or problems that bother you?	yes/no<br><br>if yes, please indicate what type of behavior problems these are. Please tick whatever behaviour applies to your dog and explain briefly in the comment box in the next question what behavior your dogs showns and in which situations:<br>Aggression (e.g. towards people, towards other dogs, when touched)<br>Fear, Phobia, Anxiety (e.g. afraid of fireworks or thunderstorms, afraid and tense while outside, afraid of people afraid of other dogs, crowded places)<br>Separation-related problems (e.g. vocalization, destruction, house soiling when alone or separate from you)<br>excessive behavior (e.g. hyperactivity, hyperreactivity, hypervigilance, cannot stop)<br>diminished behavior (e.g. depression, lack of energy)<br>repetitive behavior, stereotypic and / or compulsive behaviors (e.g. tail chasing, licking itself, turning in circles, chasing shadows)<br>travel-related problems (e.g. cannot relax in the car, barks, shows signs of car sickness)<br>other
**Surroundings**	
How many people live in the same household as your dog (including yourself)?	only me<br>2 people<br>> 2 people
Please quantify the degree of stimulation (e.g. noise, amount of traffic, people, dogs, other animals, etc.) in your dog's living surroundings.	scale 1 - 5<br>1 = very quiet<br>5 = very loud<br>I don't know<br><br>for current living surroundings<br>for first living surroundings
**Dog's history**	
What country did you get your dog from?	From the same country in which s/he lives now<br>from abroad
Did your dog have any previous owner/s (apart from the breeder)?	yes<br>no<br>I don't know
**Personality / Activity**	
Do you consider your dog to be any of the following? If yes, why? (multiple options possible)	fearful<br>neurotic<br>curious<br>sensitive<br>shy<br>uncertain<br>none of the above
How much "active" time does your dog receive per day (e.g. walks, playing in the yard / indoors, training, etc.)	< 1 hour<br>1-3 hours<br>> 3 hours<br>I don't know
When confronted with a new, unfamiliar object, how is your dog most likely to react?<br>(multiple options possible)	S/he stops and watches at a distance<br>S/he starts barking<br>S/he moves away and avoids it<br>S/he moves towards it and starts playing<br>none of the above<br>I don't know
